# Opportunities and barriers in health education and community awareness program: a qualitative study from Indian tertiary care teaching hospital

**DOI:** 10.1186/s12909-026-09563-3

**Published:** 2026-08-17

**Authors:** Rubina Saha, Debkumar Pal, Manish Taywade, Sonu H. Subba, Remya Mary John

**Affiliations:** 1 ESIC Medical College and Hospital, Hyderabad, India; 2 All India Institute of Medical Sciences, Kalyani, India; 3 All India Institute of Medical Sciences, Bhubaneswar, India; 4 All India Institute of Medical Sciences, Mangalagiri, India

**Keywords:** Health education, Community health, Undergraduate medical students, Qualitative research, Phenomenology, Community mobilization, Preventive healthcare.

## Abstract

**Background:**

Health education is an important public health intervention that promotes disease prevention, awareness generation, and healthy behavioural practices within communities. Undergraduate medical students are increasingly involved in community-based health education activities; however, they often encounter several operational and communication-related challenges during field implementation. This study explored the perceptions of undergraduate medical students regarding the challenges and opportunities in imparting health education in community settings.

**Methods:**

A phenomenological qualitative study was conducted among third-year undergraduate medical students in the Department of Community Medicine and Family Medicine at All India Institute of Medical Sciences Bhubaneswar between July 2022 and October 2022. Participants were selected through purposive sampling after attending health education training and participating in community-based health education activities. In-depth interviews were conducted using a semi-structured interview guide. Data saturation was achieved after nine interviews; additional interviews were conducted to improve data richness. Interviews were audio-recorded, transcribed verbatim, and analysed thematically using ATLAS.ti.

**Results:**

A total of fifteen undergraduate medical students participated in the study. Participants perceived health education as an important tool for improving community awareness, preventive healthcare, and medication adherence. Interactive methods such as skits, role-plays, posters, demonstrations, and local-language communication were considered effective in enhancing community engagement. Major challenges identified included language barriers, inadequate training, limited institutional support, logistical constraints, poor community participation, and excessive workload among community mobilizers. Students emphasized that collaboration with ASHA workers and community mobilizers improved rapport building and public participation. Opportunities identified included stronger faculty mentorship, better planning, culturally sensitive communication, community-centred educational strategies, and enhanced institutional and community support.

**Conclusion:**

Undergraduate medical students recognized the importance of health education in improving community health; however, several communication, organisational and logistical barriers affected its implementation in community settings. Strengthening community-oriented medical education, local-language communication skills, faculty mentorship, and collaboration with community mobilizers may improve the effectiveness and sustainability of community health education programmes.

**Supplementary Information:**

The online version contains supplementary material available at https://doi.org/10.1186/s12909-026-09563-3.

## Introduction

Health education is the process of enabling people to increase control over and improve their health. It involves communicating information to enhance knowledge, influencing attitudes, and motivating changes in behaviour [1]. The Ottawa Charter defined health promotion as enabling people to increase control over and improve their health [2]. Health education and environmental interventions are effective in-patient care, alongside pharmacological interventions [3]. Health education not only provides information about disease treatment, but it also promotes positive behaviour change among patients. This role has been vital during the Coronavirus Disease of 2019 (COVID-19) pandemic. In the early part of the pandemic, mass media played a significant role in providing health education. This included promoting the use of masks, sanitisers, and COVID-19 vaccination [4–6]. For providing health education effectively and efficiently, the knowledge and practice for the same are required, with a supervisory role of senior faculty members inside a competency-based curriculum in medical education [3, 7].

The necessary practical communication skills can be improved among medical undergraduate students by providing a practical teaching-learning session with field practice [8]. The evidence-based literature is somewhat silent regarding undergraduates’ perceptions of health education sessions in their curriculum, whereas the importance of health education remains immense in our country [7]. The **aim** was to explore the various challenges undergraduate students face during health education sessions at the community level and to scope out ways to overcome them. This study also examined the opportunities and necessities they felt were needed while imparting health education in a community set-up.

## Methodology

This qualitative study with a phenomenological approach was conducted in the Department of Community Medicine and Family Medicine at All India Institute of Medical Sciences Bhubaneswar between July 2022 and October 2022. Ethical approval was obtained from the Institutional Ethics Committee of AIIMS Bhubaneswar [IEC No. T/IM-NF/CM&FM/22/]. The study participants were third-year undergraduate medical students who had attended clinical postings in Community and Family Medicine, received brief training in health education, and participated in delivering health education sessions in community settings within the previous three months. This duration was considered to minimize recall bias and facilitate better recollection of field experiences.

A semi-structured interview guide (Supplementary File 1) was developed by three authors and validated by four experts experienced in undergraduate health education training and community-based health promotion activities, which was pilot tested in two students. The experts were selected because of their prior involvement in similar training programmes within the same institution and their experience in mentoring undergraduate medical students during community health education activities.

A phenomenological qualitative design was chosen to explore the lived experiences of undergraduate medical students regarding challenges and opportunities encountered while imparting health education in community settings. Purposive sampling was used to recruit participants who had attended the training programme and actively participated in community health education delivery.

All students underwent training under the supervision of the same faculty members to maintain uniformity in the training process and reduce observer-related variation. Since all participants belonged to the same academic background and institution, the possibility of response variability due to educational differences was considered minimal.

In-depth interviews were conducted face-to face, after students had completed their field exposure to health education activities in the rural field practice area of the institution. The interviews followed a post-positivist paradigm approach, allowing openness to participants’ experiences and emerging perspectives. Each interview was conducted in the presence of two researchers, one male and one female from the authors, who were designated trainers to the students, bearing an experience of three and four years respectively in the field, where one researcher facilitated the interview while the other observed and documented non-verbal cues, thereby enhancing methodological rigor and minimizing observer bias. All the interviews were conducted in the institution at working hours.

The sample size was guided by the principle of thematic saturation, which is widely accepted in phenomenological qualitative research. Saturation was achieved after nine in-depth interviews, as no substantially new themes emerged in subsequent interviews. However, additional interviews were conducted to improve the richness, credibility, and confirmability of the findings, and the total number of interviews was fifteen.

All interviews were audio-recorded, transcribed verbatim, and translated into English where necessary by three authors. The transcripts were validated through member checking to ensure accuracy and credibility of the participants’ responses. Data analysis was performed using ATLAS.ti to identify themes, sub-themes, codes, and supporting quotations through thematic analysis.

## Results

A total of fifteen participants were interviewed for this study. The mean age of the undergraduate students included in the study was 20.7 years (SD 0.7). The interview was conducted with seven male students and eight female students. The mean duration of IDIs was 13.2 min, with the longest period of 24 min and 50 s and the shortest period of 6 min and 16 s (see Table 1).

**Table 1:** Distribution of baseline characteristics of undergraduate students who participated in the In-depth interview(*N* = 15)

Characteristics	Mean(SD)/*n*(%)
Age	20.73(± 0.70)
Gender	
Male	7(46.67)
Female	8(53.33)
Average time of Interview(Mins: Secs)	13:17(± 5:75)
Highest time Interview (Mins: Secs)	24:50
Lowest time of interview (Mins: Secs)	6:16

Further, the coding and theme generation, along with sentiment analysis, can be seen in Supplementary file 2.

### Health education: a significant public health intervention for prevention and creating awareness

Participants viewed health education as a vital public health intervention that raises awareness of common illnesses. Effective health awareness stems from understanding community needs and providing valuable health-promotion insights. Health education can guide communities and disseminate awareness on disease prevention (See Supplementary file 3: Table 1).

One participant stated:

“Health education, for me, is a broad concept that involves spreading information regarding disease and methods of prevention of disease, how the disease spreads in the community…” (Respondent 1).

Students also viewed health education as a tool for community empowerment and awareness generation. They emphasized that even simple health-related information could positively influence individuals and families.

A participant highlighted:

“Simple information that may seem obvious to us can be unknown to others, so spreading awareness of correct and accurate information is crucial.” (Respondent 3).

Another participant emphasized that health education should extend beyond healthcare settings and reach the wider community:

“It is not only restricted to people of the health community; it should be spread to everyone…” (Respondent 2).

Participants further believed that health education contributes to improved medication adherence, prevention of complications, and better health outcomes among the community.

### Innovative health education methods can increase community involvement and medication adherence, improving health outcomes

Students believe that unique methods can boost health awareness, like using techniques such as role-play and effective doctor-patient communication. Making health education interactive engages the community, enhancing the efficiency of health promotion. Embracing health education fosters cultural awareness and positively impacts society. They also noted that improved health promotion could enhance patient medication adherence. (See Supplementary file 3: Table 1)

One respondent explained:

“We can also use role-playing, posters, and other visuals in the community to help them understand.” (Respondent 3).

Participants also highlighted the usefulness of local-language communication and community-based campaigns in improving public participation and comprehension.

“We can provide health education through charts and demonstrations in the local language, and we have to make it more interesting rather than imparting it stereotypically.” (Respondent 7).

Another participant shared their experience of using entertainment-based approaches:

“We put on a skit at a school to spread the word about geriatric problems. We made it entertaining so the kids would pay attention…” (Respondent 12).

Students additionally perceived mass media and mobile-based communication as potential tools for strengthening health promotion activities.

### Health education as a catalyst for community engagement

Participants believed that health education activities strengthen community participation and improve rapport between healthcare providers and the community. They emphasised the role of family involvement, community trust, and public engagement in successful health promotion. One participant described the positive response received during community activities:

“The community responded positively to our promotional activity. They were interactive in their questioning and engaged well.” (Respondent 5).

### Everyone has a different responsibility to fulfil while imparting health education

Students should engage empathetically with their communities to promote health. They see their institute as vital in health promotion through rural and urban training centres. In outpatient areas, hospitals can display easy-to-understand IEC (Information, Education, Communication) banners for patients. Peripheral centres ensure early treatment and the prevention of complications. The institution guides students in health education and provides reference materials. Qualified doctors and experts can further advance health education. (See Supplementary file 3: Table 2)

A participant stated:

“Our institute plays a critical role in providing health education. Peripheral centres have been established to educate the community on health issues.” (Respondent 9).

Students also highlighted the role of community mobilizers in establishing rapport and increasing participation in health education programmes.

“The responsibility of community mobilizers is to motivate the committee members to come forward and participate in the health education programme.” (Respondent 13).

Another participant added:

“The community mobilizers can help us in building a good rapport with the community.” (Respondent 5).

The participants identified multiple interconnected challenges that hindered the effective delivery of health education in community settings. The major barriers included communication and language difficulties, inadequate training and institutional support, logistical and resource-related constraints, limited community participation, and challenges faced by community mobilizers. (See Table 2).

**Table 2:** Thematic analysis of undergraduates’ insight on various barriers and opportunities of health education in improving community health (*N* = 15)

Theme	Sub-theme	Codes
Personal and Communication Barriers	Language and communication challenges	Local-language barrier, Difficulty conveying health messages, Communication gap, Limited interpersonal communication skills
	Inadequate training and lack of confidence	Insufficient practical training, Lack of preparedness, Low confidence in public interaction, Inadequate health education exposure
Systemic and Resource-related Challenges	Financial, manpower, and logistical limitations	Shortage of manpower, Limited financial support, Transportation barriers, Equipment unavailability, Logistic constraints, Time limitations
	Poor planning, coordination, and institutional support	Weak programme planning, Lack of coordination, Inadequate organisational support, Restricted supervision, Poor contingency preparedness
Community-level Challenges in Health Education	Limited community participation and sociocultural barriers	Community disinterest, Sociocultural resistance, Poor public engagement, Family-level non-support, Low health awareness
	Challenges in public mobilization and programme implementation	Delayed crowd mobilization, Difficulty gathering participants, Implementation barriers, Weather-related disruption, Low participation turnout
Opportunities for Strengthening Health Education	Interactive and community-centered educational strategies	Skit-based education, Community-oriented learning, Interactive teaching methods, Use of audio-visual aids, Community need-based interventions
	Professional mentorship and institutional facilitation	Structured faculty guidance, Advance planning, Professional supervision, Confidence-building support, Institutional credibility
	Community support, incentives, and collaborative engagement	Incentive-based participation, Free healthcare services, Collaboration with ASHA workers, Community reinforcement strategies, Sustainable community engagement

### The language barrier

One of the most frequently reported barriers was the language and communication gap between students and the community. Participants explained that lack of fluency in the local language reduced their confidence and limited their ability to effectively communicate health messages. This barrier affected both active participation and audience comprehension during health education activities (See Supplementary file 3: Fig. 1).

“I faced a challenge in effectively communicating my message as the individuals were unfamiliar with the Hindi language.” (Respondent 3).

“I participated in the skit as an actor but only held a chart because I was not fluent in the local language.” (Respondent 4).

Students also perceived that communication barriers reduced community understanding and engagement during health promotion sessions.

“Unfortunately, fewer people participated and could not understand us as well as they would have if they had listened to us properly.” (Respondent 7).

Participants believed that local-language communication and culturally sensitive interaction were essential for effective health education delivery and community engagement.

### Inadequate training and organisational support

Participants expressed that they were insufficiently prepared to conduct health education activities in community settings. Many students felt that health education received limited emphasis during undergraduate medical training and that they lacked practical exposure, communication skills, and strategies for community engagement.

“The institute is facing challenges because health education is not considered a core part of medicine, and there is a need for more understanding of its role.” (Respondent 11).

Students also highlighted inadequate planning and preparedness while organizing community-based activities.

“We should have a contingency plan in case of any issues caused by nature.” (Respondent 8).

These findings indicate the need for stronger faculty mentorship, structured training, and greater institutional prioritization of health education within medical curricula.

### Logistical and resource-related challenges

Students reported several operational barriers that affected the implementation of health education activities. These included transportation difficulties, inadequate equipment, weather-related disruptions, and poor coordination.

“We encountered logistical issues in transporting all necessary equipment, such as a microphone and speaker.” (Respondent 7).

“It suddenly started raining. Due to this, some related people could not make it on time.” (Respondent 7).

Participants further explained that inadequate communication and poor planning reduced participation and affected programme organization.

“It’s unfair to expect people to show up without informing them at least a day before.” (Respondent 7).

The findings suggest that insufficient logistical support and poor coordination significantly limited the effectiveness of community health promotion activities.

### Community participation and mobilization challenges

Another major challenge identified by participants was poor community participation and limited public interest in health education programmes. Students explained that motivating people to attend and actively engage during sessions was difficult.

“Community members did not appear interested in our training.” (Respondent 8).

Students believed that stronger community engagement strategies and better mobilization methods were required to improve participation and acceptance of health education activities.

### Challenges faced by community mobilizers

Participants highlighted that community mobilizers, particularly ASHA (Accredited Social Health Activist) workers and frontline health workers, played a central role in facilitating health education activities within the community. Students perceived them as important links between healthcare institutions and the public because they helped in mobilising people, building rapport, and improving participation during health promotion programmes. However, participants also emphasized that community mobilizers themselves faced several operational and systemic challenges that limited their ability to effectively support health education activities.

One of the major concerns raised by students was the excessive workload and shortage of manpower among community mobilizers. Participants believed that mobilizers were expected to handle multiple responsibilities simultaneously, making it difficult for them to independently organize and sustain community participation for health education programmes.

“The challenges community mobilizers are facing are that the system needs to be more staffed and resources are limited.” (Respondent 15).

Students perceived that inadequate staffing reduced the effectiveness of outreach activities and limited the mobilizers’ ability to gather people for health education sessions.

Another important issue identified was inadequate financial and institutional support for community mobilizers. Participants reported that ASHA workers and community-level health workers often lacked sufficient governmental assistance, incentives, and logistical backing to effectively carry out community mobilization activities. The lack of adequate support negatively affected motivation and the sustainability of community engagement efforts.

The participants also noted that mobilizers experienced difficulty in encouraging public participation because some community members showed limited interest in health education activities.

“The community mobilizers can help us in building a good rapport with the community.” (Respondent 5).

“The responsibility of community mobilizers is to motivate the committee members to come forward and participate in the health education programme.” (Respondent 13).

“ASHAs are less in numbers with respect to the population they are catering. (Respondent 13).

Also they are not given enough incentives according to the work they do.” (Respondent 10).

### Identified solutions observed by students while imparting health education

The student believes community engagement should focus on mass gatherings, target audiences, regular feedback, and cost-effective healthcare solutions. Using interactive methods like role-plays and skits during extracurricular activities and via technology can effectively deliver health education. Proper planning and tailored interventions can improve health outcomes. (See Table 2).

To boost participation, the community needs access to free healthcare and incentives for health activities. The institution must build student confidence, provide positive reinforcement, and motivate community involvement. Health education should focus on solutions and equality, fostering teamwork with medical institutions and MBBS students. Professional guidance is vital before students deliver health education. Top institutions like AIIMS should participate in community health promotion for overall health development. (See Supplementary file 3, Fig 2)

### Interactive and community-centred educational strategies

Participants believed that innovative and participatory teaching methods improved audience engagement and made health education more understandable and appealing to the community. Students emphasized that role-plays, skits, demonstrations, posters, charts, and audio-visual aids helped simplify complex health messages and encouraged active public participation.

“We can also use role-playing, posters, and other visuals in the community to help them understand.” (Respondent 3).

“We put on a skit at a school to spread the word about geriatric problems. We made it entertaining so the kids would pay attention…” (Respondent 12).

“We can resort to the use of skits, drama, street dramas, pamphlets, something that will entertain the crowd but also help promote health education.” (Respondent 11).

Participants also highlighted the importance of using local-language communication and culturally appropriate educational methods to improve audience understanding and participation.

“We can provide health education through charts and demonstrations in the local language, and we have to make it more interesting rather than imparting it stereotypically.” (Respondent 7).

Students believed that engaging and interactive approaches increased the effectiveness of health promotion activities and encouraged better retention of health-related information.

### Professional mentorship and institutional facilitation

Participants emphasized that stronger institutional involvement and professional guidance could substantially improve health education activities. Students felt that medical institutions should provide structured mentorship, training, logistical support, and opportunities for practical community engagement.

“Our institute plays a critical role in providing health education.” (Respondent 9).

“Peripheral centres have been established to educate the community on health issues.” (Respondent 9).

Students also believed that faculty guidance and advance planning were essential for improving programme implementation and overcoming operational barriers.

“The faculty could have provided more input to the activity.” (Respondent 8).

“We should have a contingency plan in case of any issues caused by nature.” (Respondent 8).

Participants suggested that reputed medical institutions should actively participate in community health promotion activities to strengthen preventive healthcare and improve public trust.

Students further highlighted the importance of prior planning, need assessment, and preparation before conducting health education activities in the community.

### Community support and collaborative engagement

Participants identified community engagement and collaboration with frontline health workers as important facilitators for improving health education delivery. Students perceived ASHA workers and community mobilizers as essential partners for identifying local health needs, motivating community participation, and building trust with the public (Supplementary file 3: Fig. 2).

“If we could communicate with ASHA and get to know what the community needs, it could also help…” (Respondent 8).

“The community mobilizers can help us in building a good rapport with the community.” (Respondent 5).

Students also emphasized the importance of involving families and local communities in health promotion activities.

“We can talk to families and encourage them to visit nearby hospitals or sub-centers.” (Respondent 13).

Participants believed that providing incentives, transportation, and free healthcare services could further improve participation in community health programmes.

“By providing incentives, transportation, and complimentary services through consultation through a health camp.” (Respondent 6).

Another opportunity identified by students was the use of mass media and mobile-based communication to disseminate health information more effectively.

“We can spread health education using mass media or phones.” (Respondent 13).

### Sentiment analysis

We explored the sentiments of the participants, and we found that they believed that collaborative engagement between students, medical institutions, community mobilizers, and local communities could substantially strengthen health education activities and improve community health outcomes.

The sentiment analysis showed that undergraduate medical students had an overall positive perception of health education and considered it an important tool for disease prevention, community awareness, and health promotion (see Supplementary file 2). Students expressed enthusiasm towards participating in community-based health education activities and believed that interactive methods such as skits, demonstrations, and local-language communication improved community engagement.

At the same time, participants expressed frustration regarding language barriers, inadequate training, logistical difficulties, poor community participation, and limited organisational support during field implementation. Despite these challenges, students maintained a constructive and solution-oriented attitude by suggesting better planning, comprehensive mentorship, collaboration with community mobilizers, and culturally appropriate communication strategies to improve health education delivery (See Supplementary file 3: Table 3).

## Discussion

This study discussed the roles and responsibilities of undergraduates in a health education session and the challenges and opportunities they perceived during the same. Conduct of health education sessions were added to the undergraduate medical curriculum of AIIMS Bhubaneswar since its inception. As part of the activity, the students are required to plan and conduct a health education session in the field practice area under the guidance of a senior faculty member. During the study, in-depth interviews were conducted, covering four major themes related to health education. These themes included students’ perception regarding health education, the roles and responsibilities of individuals involved in health promotion activities, the challenges various individuals face while providing health education, and the opportunities available to improve health education. Most students believed that health education is crucial to public health intervention. It encourages people to adopt a healthy lifestyle, and innovative techniques for disseminating knowledge on health can lead to improved health outcomes, such as adherence to medication [9, 10]. According to a study by Edward E. Bartlett, school health education programs can effectively influence behaviours related to specific health conditions that pose current and immediate problems, such as dental issues, adolescent pregnancy, smoking, and nutrition [11].

Health education enhances doctor-patient communication skills, fosters cultural awareness among those who plan to provide health education, and plays a vital role in improving community awareness of health. The students had a correct perception regarding the same. The fundamental principles of Health Settings include community participation, partnership, empowerment, and equity [7]. During interviews, students noted that various individuals were involved in providing adequate health education in the community. They believed it was their responsibility to communicate empathetically with the community and motivate them toward better health outcomes. The institution could assist in training students in this field and establish urban and rural health centres for regular health promotion activities [12]. They suggested that placing appropriate IEC banners in hospitals would help patients better understand their health conditions. Community mobilizers were crucial in representing and creating social influence in the community, which would bridge the gap between students and the community. Doctors and specialists should also be encouraged to participate in such activities [13]. Many students felt that their training needed to be improved to provide health education in the community, as the time devoted in the curriculum for health education was relatively less and needed improvement. Most students felt that language was the primary barrier that hindered effective health promotion activities, as they could not effectively communicate their message to the community. They lacked community support as they were perceived as outsiders. Moreover, the lack of proper planning and implementation of health education led to confusion. Many areas were not accessible due to transportation and logistical issues, which resulted in an unequal distribution of health promotional and awareness activities. Students also reported that community mobilizers were overburdened with work and paid low wages, making it difficult to devote their time to health education. Other challenges identified included social barriers, unpredictable weather conditions, a lack of human resources, and poor public mobilisation. During the discussion, the students suggested that proper guidance and training should be included in their curriculum to overcome the barriers. Incentives should be given to community mobilizers and target populations to increase their participation. Interactive methods such as role play and drama should be encouraged to make health promotional activities more interesting for the audience. Planning and coordination should be prioritised, and regular assessments and suggestions from the community should be taken to improve health promotional activities [14]. The study findings suggested that already established principles of health education should always be kept in mind during any health education session. The problems identified can be tackled using those principles [15]. With the nationwide implementation of Competency-Based Medical Education (CBME) in India, greater emphasis has been placed on early clinical exposure, communication skills, and community-based learning. CBME explicitly incorporates health education and community engagement as core competencies under the Attitude, Ethics, and Communication (AETCOM) module. However, despite these curricular advancements, several challenges in implementation have been reported, including restricted mentorship, variability in execution, and limited emphasis on experimental learning in real-world settings [16–18]. Students in our study reported self-directed learning, limited practical exposure, and challenges in real-world implementation, such as language barriers, logistical constraints, and a lack of community participation. These findings indicate that while CBME provides a structured framework, critical gaps persist at the level of field implementation, particularly in preparing students for community engagement and health education delivery. These findings provide practical insights for strengthening community-based training by integrating better planning, stakeholder engagement, and support mechanisms within medical education programs, as also highlighted in previous CBME implementation studies [16–18]. We identified a significant knowledge gap, where students were conceptually aware of health education but lacked context-specific understanding of community dynamics and behaviour change strategies. Second, a clear training gap emerged due to less emphasis under the curiculum and time constraint, with students reporting insufficient structured preparation, limited hands-on guidance, and a lack of standardised teaching methods for delivering community-based health education. Our findings bring attention to a timing gap within the undergraduate curriculum, where exposure to health education activities begins relatively late, limiting students’ preparedness and confidence during field implementation. This is particularly relevant in the context of Competency-Based Medical Education (CBME), which emphasises early clinical and community exposure.

Additionally, this study uniquely highlights the critical yet underexplored role of community mobilisers, such as ASHA workers, as key facilitators in bridging the gap between medical students and the community. However, their effectiveness is constrained by systemic challenges, including workload burden, inadequate incentives, and limited institutional support. Finally, the study identifies community mobilisation as a major implementation bottleneck, influenced by factors such as poor planning, lack of prior communication, sociocultural barriers, and low community engagement. These findings provide practical insights for strengthening community-based training by integrating better planning, stakeholder engagement, and support mechanisms within medical education programs.

### Strengths

A major strength of this qualitative study was the use of in-depth interviews, which provided rich and detailed insights into undergraduate medical students’ real-life experiences of imparting health education in community settings. The inclusion of verbatim quotations enhanced the credibility of the findings, while thematic analysis enabled systematic identification of important challenges and opportunities related to community health promotion. The study also explored perspectives regarding multiple stakeholders, including students, institutions, and community mobilizers, providing a comprehensive understanding of community-based health education.

## Limitations

The study took place with undergraduate students in the community and family medicine department. Therefore, this study’s findings may only represent some of the population of undergraduates pursuing MBBS. Most of our interviews lasted for more than fifteen minutes. The duration of some interviews was shorter than expected, with some lasting less than ten minutes. Shorter interview durations may have limited the depth of exploration of certain participant experiences and perspectives. However, repeated patterns and consistency in responses across participants supported thematic saturation and the credibility of the findings.

## Conclusion

Health education is one of the essential public interventions for community awareness and improving health outcomes. The students who participated shared their experiences imparting health education in the community and concluded that their skills, knowledge of society, and communication skills are the key challenges that require vital focus. They found that regular assessments, incentivising community mobilizers, and the target population may improve community participation and thus facilitate health promotion activity in the community.

## Supplementary Information

Supplementary Material 1.

Supplementary Material 2.

Supplementary Material 3.

## Data Availability

All data utilized in this study were collected exclusively through participant interviews. Due to confidentiality concerns and the potential risk of identifying participants, the data cannot be made publicly accessible. Nonetheless, the data may be obtained upon request from the primary author, Dr Rubina Saha, at rubina.saha11@gmail.com.
