# Supplementary material for: Opportunities and barriers in health education and community awareness program: a qualitative study from Indian tertiary care teaching hospital
Source: BMC Med Educ. 2026 Aug 17;26:1715. doi: 10.1186/s12909-026-09563-3 (PMC13695292; doi:10.1186/s12909-026-09563-3)
Supplement: Supplementary file 1 [file 12909_2026_9563_MOESM1_ESM.docx]

**Questionnaire for Interview of Medical Students**

**Topic: Opportunities and Barriers in Health Education and Community Awareness Program: A Qualitative Study from an Indian Tertiary Care Teaching Hospital**

**Demographic details**

**Name of the Participant-	Age-	Gender-**

**Time of interview conducted-**

**Part of Health Education training sessions in the Department of Community Medicine and Family Medicine in the last three months:**	Yes/ No

**Qualitative Part**

**Introduction**

Hello, I’m the interviewer (gives Name to the interviewee), and I will be conducting a study (gives study title to the interviewee). May I know your name, age, and designation (what year student), and have you been involved in imparting health education in the community setting or not?

**Consent**

I would like to conduct your interview and include it in my study based on an interview guide. Do you give your consent?

**Interview Guide**

**1. Idea about Health Education (Knowledge)**

**What is your general opinion on Health Education?**

**Probe-**

1.1 Do you think you have sufficient knowledge about the topic?

1.2 What is the need for health education?

1.3 Are you aware of various methods by which health education can be given?

1.4 What is the impact of health education, according to you, on society?

1.5 Do you think it is important?

1.6 What does it do?

1.7 Anything you would like to add?

**2. Responsibility**

**What is your opinion about various roles and responsibility of various stake holders in health education for community Awareness?**

**Probe-**

2.1 What is your role or responsibility in imparting health education?

2.2 How can you provide Health education in the community?

2.3 Do you think any other stakeholders have any role to play?

2.4 In the community, does anyone have any role to play for successful implication of health education in the community?

2.5 Do you think everyone provides health education equally

2.6 Any other views you would like to add?

**3. Experience**

**3.1 What was your experience in imparting health promotion activity during community health awareness training?**

**Probe-**

3.1.1 Have you considered becoming involved in health promotion or education?

3.1.2 Do you think you were sufficiently trained for health promotion activity?

3.1.3 Did you use any specific guidance documents that influence your work?

3.1.4 Do you consider your role is important in health promotion and health educational activity

3.1.5 What is the response of the community when you imparted health education?

3.1.6 Do you think there is enough participation from the community?

3.1.7 Do you think Health resources provided to you was enough to impart knowledge in the community awareness?

3.1.8 Do you think everyone provides health education equally ?

3.1.9 What is the role of community mobilisers?

3.1.10 What is the role of Institution?

3.1.11 How can we( Teachers) help you?

3.1.12 Do you think you get enough support from the organisations for health promotion activity?

3.1.13 What do you have to say about the community support?

**3.2 What are the challenges you have faced while imparting health education in the Community?**

**Probe-**

3.2.1 What are the challenges which you faced?

3.2.2 What are the challenges you faced due to circumstances?

3.2.3 What are the prior challenges you faced before imparting health education in the community?

3.2.4 What are the challenges you faced at the time of giving health education?

3.2.5 Do you want to add anything else?

**3.3 What are the Suggestions you would like to give through your experience so that it may help in facilitating health education given ?**

**Probe-**

3.3.1 What is your opinion about the changes which can be made for betterment of the health promotion in community awareness?

3.3.2 Do you think you could have improved the health education in a better way, if yes then how?

3.3.3 How can you improve the support from these organisations(state, centre, community)

3.3.4 How can you improve the community support?

3.3.5 Any other suggestions you would like to give?
