# Supplementary material for: Opportunities and barriers in health education and community awareness program: a qualitative study from Indian tertiary care teaching hospital
Source: BMC Med Educ. 2026 Aug 17;26:1715. doi: 10.1186/s12909-026-09563-3 (PMC13695292; doi:10.1186/s12909-026-09563-3)
Supplement: Supplementary file 2 [file 12909_2026_9563_MOESM2_ESM.docx]

**Coding Tree Derived from Thematic Analysis ( Refined by ChatGPT)**

**Theme 1: Perceived Importance of Health Education**

**Sub-theme 1.1: Health education as a preventive public health strategy**

| **Quotation** | **Code** |
| --- | --- |
| “Health education, for me, is a broad concept that involves spreading information regarding disease and methods of prevention of disease…” (R1) | Disease prevention awareness |
| “…how the disease spreads in the community…” (R1) | Community disease literacy |
| “It’s essential to focus on prevention, early treatment, and prevention of complications…” (R3) | Preventive healthcare promotion |
| “Health education plays a significant role in preventing diseases…” (R3) | Public health advocacy |

**Sub-theme 1.2: Health education as a tool for awareness and empowerment**

| **Quotation** | **Code** |
| --- | --- |
| “Simple information that may seem obvious to us can be unknown to others…” (R3) | Knowledge dissemination |
| “It should be spread to everyone…” (R2) | Community-wide awareness |
| “…spreading awareness of correct and accurate information is crucial.” (R3) | Health literacy enhancement |
| “It should not only be given to those who need it, but also to those who can propagate it further.” (R2) | Community empowerment |

**Theme 2: Innovative Approaches for Health Promotion**

**Sub-theme 2.1: Interactive and participatory educational approaches**

| **Quotation** | **Code** |
| --- | --- |
| “We can also use role-playing, posters, and other visuals…” (R3) | Role-play based learning |
| “We made it entertaining so the kids would pay attention…” (R12) | Entertainment-based education |
| “We can resort to the use of skits, drama, street dramas…” (R11) | Interactive community education |
| “We can provide health education through charts and demonstrations…” (R7) | Demonstration-based teaching |

**Sub-theme 2.2: Use of culturally appropriate and mass-media communication**

| **Quotation** | **Code** |
| --- | --- |
| “…through charts and demonstrations in the local language…” (R7) | Local-language communication |
| “We can spread health education using mass media or phones.” (R13) | Digital and mass-media outreach |
| “…show informative videos at local events like melas…” (R6) | Community campaigns |
| “…make it more interesting rather than imparting it stereotypically.” (R7) | Audience-centred communication |

**Theme 3: Health Education as a Catalyst for Community Engagement**

**Sub-theme 3.1: Family and community participation in health promotion**

| **Quotation** | **Code** |
| --- | --- |
| “The community responded positively to our promotional activity.” (R5) | Positive community participation |
| “They were interactive in their questioning and engaged well.” (R5) | Active public engagement |
| “…we can talk to families and encourage them…” (R13) | Family-level engagement |
| “…helping our community live better!” (R12) | Community empowerment |

**Sub-theme 3.2: Building trust and social connectedness through health education**

| **Quotation** | **Code** |
| --- | --- |
| “The community mobilizers can help us in building a good rapport with the community.” (R5) | Rapport building |
| “The responsibility of community mobilizers is to motivate…” (R13) | Community engagement |
| “…learn precisely what conditions the people in the town might be suffering from.” (R10) | Community need assessment |
| “…we had targeted a different population…” (R14) | Community outreach adaptation |

**Theme 4: Stakeholder Responsibilities in Health Education**

**Sub-theme 4.1: Role of medical students in community health promotion**

| **Quotation** | **Code** |
| --- | --- |
| “We, being undergraduates, can impart health education to the community.” (R7) | Student-led health education |
| “…educating the people at our level and in a simple language is essential.” (R3) | Simplified community communication |
| “We interview a family specifically…” (R10) | Community outreach participation |
| “…help them understand.” (R3) | Community engagement responsibility |

**Sub-theme 4.2: Empathy-driven community interaction and mobilization**

| **Quotations** | **Codes** |
| --- | --- |
| “The community responded positively to our promotional activity. They were interactive in their questioning and engaged well.” (Respondent 5) | Positive community engagement |
| “We asked them what the take-home message would be… and they responded very well.” (Respondent 5) | Reciprocal communication |
| “We interview a family specifically through which we learn precisely what conditions the people in the town might be suffering from.” (Respondent 10) | Empathetic understanding |
| “We can provide health education through charts and demonstrations in the local language…” (Respondent 7) | Culturally sensitive communication |

**Sub-theme 4.3: Institutional support for health education**

| **Quotation** | **Code** |
| --- | --- |
| “Our institute plays a critical role in providing health education.” (R9) | Institutional health promotion support |
| “Peripheral centres have been established…” (R9) | Community outreach infrastructure |
| “Our doctors provide health education…” (R9) | Faculty-led health education |
| “The faculty could have provided more input…” (R8) | Need for faculty mentorship |

**Sub-theme 4.4 : Institutional infrastructure and health promotion resources**

| **Quotations** | **Codes** |
| --- | --- |
| “Peripheral centres have been established to educate the community on health issues.” (Respondent 9) | Peripheral outreach infrastructure; Community outreach services |
| “Our OPDs have multiple IEC banners to raise awareness among patients.” (Respondent 9) | IEC material utilization; Visual health promotion tools |
| “Our doctors provide health education through active and passive counselling to ensure that patients are well-informed.” (Respondent 9) | Counselling-based health education; Provider-led awareness |
| “When patients go to the hospital, the doctor can explain the different programs and facilities available to them.” (Respondent 3) | Healthcare-based patient education; Information dissemination |
| “We can use role-playing, posters, and other visuals in the community to help them understand.” (Respondent 3) | Educational visual aids; Interactive IEC materials |
| “We can provide health education through charts and demonstrations in the local language.” (Respondent 7) | Demonstration-based education; Local-language educational materials |
| “We can share it during community meetings, post helpful posters, and even show informative videos at local events like melas.” (Respondent 6) | Community-based IEC dissemination; Multimedia health promotion |
| “The faculty could have provided more input to the activity.” (Respondent 8) | Need for institutional mentorship; Faculty support gap |

**Sub-theme 4.5: Role of community mobilizers**

| **Quotation** | **Code** |
| --- | --- |
| “The responsibility of community mobilizers is to motivate…” (R13) | Grassroot mobilization |
| “…help us in building a good rapport…” (R5) | Community linkage facilitation |
| “…get to know what the community needs…” (R8) | Community need identification |
| “…participate in the health education programme.” (R13) | Participation facilitation |

**Theme 5: Barriers to Effective Health Education**

**Sub-theme 5.1: Personal and communication barriers**

| **Quotation** | **Code** |
| --- | --- |
| “I faced a challenge in effectively communicating…” (R3) | Communication barrier |
| “…I was not fluent in the local language.” (R4) | Language discordance |
| “…could not understand us as well…” (R7) | Limited audience comprehension |
| “We should have been trained better…” (Implied across interviews) | Inadequate practical training |

**Sub-theme 5.2: Systemic and resource-related challenges**

| **Quotation** | **Code** |
| --- | --- |
| “…logistical issues in transporting all necessary equipment…” (R7) | Logistic constraints |
| “…some related people could not make it on time.” (R7) | Participation disruption |
| “…resources are limited.” (R15) | Resource limitation |
| “…health education is not considered a core part of medicine…” (R11) | Institutional undervaluation |

**Sub-theme 5.3: Community-level implementation challenges**

| **Quotation** | **Code** |
| --- | --- |
| “Community members did not appear interested…” (R8) | Low community interest |
| “…fewer people participated…” (R7) | Poor participation turnout |
| “…difficulty gathering the crowd…” (Implied) | Mobilization difficulty |
| “…social barriers…” (Narrative finding) | Sociocultural resistance |

**Theme 6: Opportunities for Strengthening Health Education**

**Sub-theme 6.1: Interactive and community-centred educational strategies**

| **Quotation** | **Code** |
| --- | --- |
| “We can resort to the use of skits, drama…” (R11) | Interactive educational strategy |
| “…health promotion activity through a skit.” (R10) | Community-centred learning |
| “…make it entertaining…” (R12) | Engagement-oriented education |
| “…through charts and demonstrations…” (R7) | Visual learning facilitation |

**Sub-theme 6.2: Professional mentorship and institutional facilitation**

| **Quotation** | **Code** |
| --- | --- |
| “The faculty could have provided more input…” (R8) | Need for faculty guidance |
| “We should have a contingency plan…” (R8) | Advance programme planning |
| “…professional guidance is important…” (Narrative) | Structured mentorship |
| “…all top educational institutions should engage…” (Narrative) | Institutional collaboration |

**Sub-theme 6.3: Community support and collaborative engagement**

| **Quotation** | **Code** |
| --- | --- |
| “By providing incentives, transportation, and complimentary services…” (R6) | Incentive-based participation |
| “…communicate with ASHA and get to know what the community needs…” (R8) | Collaboration with community workers |
| “…encourage them to visit nearby hospitals…” (R13) | Community healthcare linkage |
| “…continued reinforcement…” (Narrative) | Sustained community engagement |

**Sentiment Analysis of Undergraduate Medical Students Regarding Health Education**

The narratives showed mostly positive views of health education among undergraduates, with some negative comments about barriers and challenges. Overall, students were enthusiastic, socially responsible, and hopeful about health education's role in improving community health.

**1. Positive Sentiment Toward Health Education**

Most participants expressed strong positive attitudes toward health education and viewed it as an important preventive public health strategy. Students believed that health education improves awareness, empowers communities, promotes healthy behaviour, and prevents diseases.

**Dominant Positive Emotions Identified**

- Enthusiasm
- Social responsibility
- Motivation
- Empathy
- Optimism
- Sense of contribution
- Professional commitment

**Representative Quotations**

“Health education plays a significant role in preventing diseases…” (Respondent 3)

“It should be spread to everyone…” (Respondent 2)

“We, being undergraduates, can impart health education to the community.” (Respondent 7)

“The community responded positively to our promotional activity.” (Respondent 5)

**Positive Sentiment Codes**

- Preventive healthcare advocacy
- Community empowerment
- Student engagement
- Motivation for public service
- Positive community interaction
- Confidence in health promotion
- Interest in community medicine

**Interpretation**

Students demonstrated a strong belief that health education can create meaningful improvements in community health outcomes. They expressed satisfaction when communities responded positively and felt motivated to contribute to public health promotion.

**2. Neutral and Reflective Sentiment**

Some participants expressed analytical and reflective opinions regarding the implementation of health education. These responses reflected awareness of practical realities and the need for improvement rather than emotional positivity or negativity.

**Dominant Neutral/Reflective Emotions**

- Awareness
- Critical reflection
- Professional insight
- Practical understanding

**Representative Quotations**

“We should have a contingency plan in case of any issues caused by nature.” (Respondent 8)

“If we could communicate with ASHA and get to know what the community needs…” (Respondent 8)

“We interview a family specifically through which we learn precisely what conditions the people in the town might be suffering from.” (Respondent 10)

**Neutral/Reflective Codes**

- Need assessment
- Programme planning
- Community-oriented approach
- Collaborative learning
- Reflective practice

**Interpretation**

Students demonstrated insight into the operational and contextual aspects of community health education. Their responses indicated a willingness to improve future activities through better planning and collaboration.

**3. Negative Sentiment Related to Challenges and Barriers**

Participants also expressed frustration and concern regarding barriers encountered during health education activities. Negative sentiments were mainly associated with communication difficulties, inadequate training, limited resources, poor participation, and insufficient institutional support.

**Dominant Negative Emotions**

- Frustration
- Lack of confidence
- Disappointment
- Anxiety
- Helplessness
- Dissatisfaction

**Representative Quotations**

“I faced a challenge in effectively communicating my message…” (Respondent 3)

“I was not fluent in the local language.” (Respondent 4)

“Community members did not appear interested in our training.” (Respondent 8)

“The system needs to be more staffed and resources are limited.” (Respondent 15)

**Negative Sentiment Codes**

- Communication anxiety
- Language-related insecurity
- Perceived inadequate preparation
- Resource frustration
- Institutional dissatisfaction
- Community disengagement

**Interpretation**

Negative sentiments emerged primarily from operational barriers rather than from dislike of health education itself. Students appeared willing to participate in health promotion but felt constrained by inadequate preparation, poor logistics, and limited institutional and community support.

**Overall Sentiment Pattern**

| **Sentiment Category** | **Overall Pattern** |
| --- | --- |
| Positive Sentiment | Predominant |
| Neutral/Reflective Sentiment | Moderate |
| Negative Sentiment | Context-specific and barrier-related |

**Overall Interpretation**

The sentiment analysis indicates that undergraduate medical students hold a highly favourable attitude towards health education and strongly recognise its importance in improving community health. Despite several implementation-related challenges, students remained motivated and solution-oriented. Their narratives reflected a desire for better training, institutional support, and community collaboration to enhance the effectiveness of health education activities. The findings suggest that strengthening practical exposure, communication skills, and mentorship during undergraduate training could further improve students’ confidence and participation in community health promotion initiatives.

**Sentiment Analysis of Undergraduate Medical Students Regarding Health Education**

The qualitative narratives reflected a predominantly **positive and constructive sentiment** among undergraduate medical students toward health education, although several **negative sentiments related to barriers and implementation challenges** were also identified. Overall, students demonstrated enthusiasm, social responsibility, and optimism regarding the role of health education in improving community health.
