# Supplementary material for: Opportunities and barriers in health education and community awareness program: a qualitative study from Indian tertiary care teaching hospital
Source: BMC Med Educ. 2026 Aug 17;26:1715. doi: 10.1186/s12909-026-09563-3 (PMC13695292; doi:10.1186/s12909-026-09563-3)
Supplement: Supplementary file 3 [file 12909_2026_9563_MOESM3_ESM.docx]

*Table 1: Thematic analysis of undergraduates' insight on the role of health education in improving community health (N=15)*

| **Themes** | **Sub-themes** | **Codes** |
| --- | --- | --- |
| Perceived importance of health education | Health education as a preventive public health strategy | Disease prevention awareness, Community disease literacy, preventive healthcare promotion, public health advocacy |
|  | Health education as a tool for awareness and empowerment | Knowledge dissemination, community-wide awareness, health literacy enhancement, community empowerment |
| Innovative Approaches for Health Promotion | Interactive and participatory educational approaches | Role-play based learning, Entertainment based education, interactive community education. |
|  | Use of culturally appropriate and mass-media communication | Local-language communication, Digital and mass-media outreach, community campaigns, audience- centred communication |
| Health Education as a Catalyst for Community Engagement | Family and community participation in health promotion | Positive community participation, active public engagement, family-level engagement, community empowerment. |
|  | Building trust and social connectedness through health education | Rapport building, Community engagement, Community need assessment, community outreach adaptation |
| Impact of Health Education on Health Outcomes | Promotion of healthy behaviour and preventive practices | Healthy lifestyle promotion, Preventive health practices, Improved health-seeking behaviour, Long-term health improvement, Cultural sensitivity in healthcare |
|  | Improving medication adherence and treatment compliance | Medication adherence awareness, Rational drug-use education, Treatment compliance, Prevention of treatment default, Access to healthcare information |

The thematic analysis revealed that undergraduate medical students perceived health education as an important public health intervention for disease prevention, awareness generation, and community empowerment. Participants emphasized that health education improves health literacy and promotes preventive healthcare practices within communities. Students highlighted the importance of innovative and participatory approaches such as role-plays, skits, demonstrations, and culturally sensitive local-language communication to improve community engagement and understanding.

The findings also demonstrated that health education strengthened community participation, family involvement, trust-building, and social connectedness between healthcare providers and the community. Furthermore, students believed that effective health education could positively influence healthy behaviours, preventive practices, medication adherence, and treatment compliance, thereby contributing to long-term improvement in community health outcomes.

*Table 2: Thematic analysis of undergraduates' insight on the role and responsibility of different stakeholders in conducting a health education session(N=15)*

| **Theme** | **Sub-theme** | **Codes** |
| --- | --- | --- |
| **Role of Medical Students in Community Health Promotion** | Student participation in community awareness activities | Student-led awareness campaigns, Peer-to-community education, Health promotion participation, Community outreach involvement |
|  | Empathy-driven community interaction and mobilization | Empathetic communication, Community rapport, Public engagement, Community mobilization, Social responsibility |
| **Institutional Support for Health Education** | Faculty mentorship and professional guidance | Faculty supervision, Professional mentorship, Skill development support, Evidence-based guidance, Capacity building |
|  | Institutional infrastructure and health promotion resources | IEC resource availability, Health promotion infrastructure, Peripheral outreach centres, Preventive healthcare support, Institutional commitment |
| **Community Mobilization and Partnership Building** | Role of community mobilizers in enhancing participation | Grassroot mobilization, Community participation facilitation, Motivational support, Public engagement coordination |
|  | Bridging communication between healthcare systems and communities | Healthcare-community linkage, Community outreach coordination, Social influence on participation, Institutional-community collaboration |

The thematic analysis highlighted the significant role of undergraduate medical students in promoting community health through active participation in health awareness activities and community outreach programmes. Students perceived themselves as important contributors to health promotion and emphasized the value of empathetic communication, rapport building, and public engagement while interacting with community members. They believed that student-led awareness activities, peer-to-community education, and community mobilization could improve public understanding of preventive healthcare practices and strengthen community participation in health promotion initiatives.

Participants also emphasized the importance of institutional and community-level support in strengthening health education activities. Faculty mentorship, professional guidance, training support, and availability of IEC materials were considered essential for improving students’ confidence and effectiveness during community sessions. In addition, community mobilizers were viewed as key stakeholders in facilitating public participation, bridging communication gaps between healthcare systems and communities, and improving coordination during outreach activities. Students believed that stronger collaboration between institutions, healthcare workers, and community mobilizers could significantly enhance the effectiveness and sustainability of community health education programmes.

*Table3: Sentiment analysis of the participants regarding health education in a community setting(N=15)*

| **Sentiment Category** | **Overall Pattern** |
| --- | --- |
| Positive Sentiment | Predominant |
| Neutral/Reflective Sentiment | Moderate |
| Negative Sentiment | Context-specific and barrier-related |

The sentiment analysis indicates that undergraduate medical students possess a highly favourable attitude toward health education and strongly recognize its importance in improving community health. Despite experiencing several implementation-related challenges, students remained motivated and solution-oriented. Their narratives reflected a desire for better training, institutional support, and community collaboration to enhance the effectiveness of health education activities. The findings suggest that strengthening practical exposure, communication skills, and mentorship during undergraduate training could further improve students’ confidence and participation in community health promotion initiatives.

*Figure 1: Verbatim used for challenges identified by students in imparting health education in the community, based on their frequency of use.*

*Figure 2: Verbatim used for suggestions by students for improvement of health education in the community according to their frequency of use.*
